# Integrated approaches for plastic waste management

**DOI:** 10.3389/fmicb.2024.1426509

**Published:** 2024-09-26

**Authors:** Rajkumar D. Kherdekar, Avinash B. Ade

**Affiliations:** Department of Botany, Savitribai Phule Pune University, Pune, India

**Keywords:** plastic, polyethene, poly(propene), microbes, degradation, biodegradation

## Abstract

Plastic pollution is the challenging problem of the world due to usage of plastic in daily life. Plastic is essential for packaging food and other goods and utensils to avoid the risk of microbial attack. Due to its hydrophobic nature, it is used for wrapping as laminates or packaging liquid substances in pouches and sachets. The tensile strength of the plastic is more therefore it is used for manufacturing carrying bags that can bear heavy loads. Plastic is available in various forms as per the requirements in our daily life. Annually millions to trillions of polyethene carry bags are being manufactured and utilized throughout the world. The plastic requires millions of years for natural degradation. The physical and chemical processes are able to degrade plastic material at the meager level by 200 to 500 years in natural conditions. Many industries focus on recycling of plastic. Biodegradation is a comparatively slow and cheaper process that involves microbes. To dispose of plastic completely there is a need of an integrated process in which all the possible methods of disposal are involved and used sustainably so that minimum depletion occurs to the livestock and the environment. In the current review, we could try to emphasize the intricate nature of plastic polymers, pollution caused by it and possible mitigation strategies for plastic waste management.

## 1 Introduction

After industrialization, the whole world is facing the problem of plastic pollution. Plastic pollution contributes a significant part in solid waste. The problem of plastic pollution is raised due to its utilization which has unique molding properties to transform into several shapes (Seymour, 1989). Plastic is a broad term encompassing a wide range of polymers utilized in various industries and everyday life (Zhou et al., 2022). Plastic products have been used in daily life activities like aviation, construction, and chemical manufacturing (Banu et al., 2020; Chia et al., 2020; Lu et al., 2023). Although plastics are inexpensive, possess excellent properties, and are easy to manufacture, giving them a significant market share, they are resistant to degradation (Ali et al., 2021; Miloloza et al., 2022). Annual global plastic production has recently surpassed 380 million metric tons (Paco et al., 2017), however, only 60% of the 250 million metric tons of plastic waste is generated yearly (Skoczinski et al., 2021). Under a scenario with a moderate rate of waste-to-debris conversion, the total amount of plastic waste in the ocean is predicted to increase from 50 million metric tons in 2015 to 150 million metric tons by 2025. The accumulation of plastic in the environment happens when the influx of plastic pollution into an area surpasses the pace of natural removal processes or cleanup efforts. Plastics are highly persistent, with natural removal rates spanning decades to centuries (Chamas et al., 2020). Plastic production is increased daily, as per previous estimates by 2016 about 9 and 23 million metric tons of plastic waste entered into rivers, lakes, and oceans worldwide. In the terrestrial environment, 13 and 25 million metric tons of plastic waste entered (Borrelle et al., 2020; Lau et al., 2020). Plastic is continuously accumulated on the ocean surface over the past 60 years in the marine environment (Ostle et al., 2019). The complicated nature of plastic pollution gets changed as it undergoes weathering in the environment, and the potential for significant and difficult-to-reverse effects could result from its continued accumulation (Jahnke et al., 2017). Analysis corroborates that plastic pollution aligns with the characteristics of a planetary boundary threat (Arp et al., 2021). Remote coastlines and the ocean’s surface are recognized as major global accumulation zones for floating plastic debris. It involves the five gyres located in the North and South Pacific, North and South Atlantic, and the Indian Ocean. It is estimated that the ocean’s surface currently contains less than 0.3 million metric tons of plastic (Van Sebille et al., 2020). It is anticipated that the majority of plastic particles will eventually settle on the seafloor, and a significant portion remain suspended in the water column (Tekman et al., 2020). A recent study indicates that oxygen and nutrients supply to deep-sea fauna near-bed thermohaline currents became the site of plastic deposition into hotspots of seabed biodiversity (Kane et al., 2020). The placid, dark, cold environment of the seafloor is not conducive to further degradation therefore plastic accumulates on the seafloor at a large scale (Tekman et al., 2017; Brandon et al., 2019). In view of this, the following questions need to be addressed.

1. What are the types of plastic involved in pollution and needs to dispose?
2. What is the impact of plastic pollution on plants and animals?
3. What are the current methods available for plastic disposal?
4. Whether the integrated approach of all the possible methods is appropriate for the disposal of plastic?
5. What are the difficulties in the management of plastic waste?
6. What are the confirmation methods for the degradation of plastic waste?

To resolve these questions, the review is going to take an account of the types of plastic and the management of the plastic waste using different methods in order to follow integrated approach.

## 2 Types of plastics

Plastics are divided into thermoplastics and thermosetting plastics (Alauddin et al., 1995). These include a variety of polymers depending on the usage. Poly(1-phenylethene-1,2-diyl), polycarbonate, poly(propene), nylon, poly(1-chloroethylene), poly(ethyl benzene-1,4-dicarboxylate), polyethylene, and poly(1,1,2,2-tetrafluoroethylene) come under the thermoplastics group, and ethylurea is an example of thermosetting plastic (Smith, 1964).

Plastics are available in two forms biodegradable and non-biodegradable. Poly(hexano-6-lactone), poly(lactic acid), poly(butylene succinate-ran-butylene adipate), poly(butylene succinate-*co*-adipate), and poly(3-hydroxybutyrate-*co*-3-hydroxyvalerate) are biodegradable. Non-biodegradable plastics are polyethene, poly(ethyl benzene-1,4-dicarboxylate), poly(propene), poly(1-phenylethene-1,2-diyl), and poly(1-chloroethylene) (Urbanek et al., 2018). Some of the important types of plastic are described as follows.

### 2.1 Nylon

Nylon is a semi-crystalline artificial polymer of the polyamide family (Winnacker, 2017). It is based on aliphatic or semi-aromatic synthetic poly-amido-saccharides (Palmer, 2005). It is usually referred to as polyamide as it consists of repeating units linked by an amide bond. Nylon was originally appeared in the market in the year 1938, with poly(azanediyladipoylazanediylhexane-1,6-diyl) being the primary variant. Toothbrush bristles were the first product of nylon fibers which were released by DuPont. However, it was the introduction of nylon stockings with substantially superior tear-resistance than the natural fiber stockings (Shakiba et al., 2021). Nylons are of three types, i.e., poly(dodecano-12-lactam), poly(azanediyladipoylazanediylhexane-1,6-diyl), and poly[azanediyl(1-oxohexane-1,6-diyl)].

Nylon is a multipurpose material and comes under the thermoplastic group. By melting, molding, and cooling, film and fibers of nylon are produced (Winnacker and Rieger, 2016; Coates and Getzler, 2020). Nylon has been broadly used for the production of food packaging films, molded plastics, and clothes (Winnacker et al., 2019). In 2016 castor oil was used as raw material for nylon production. Nylons are synthesized from diamines and petroleum-derived dicarboxylic acids by condensation polymerization or ring-opening polymerization reaction (McKeen, 2017). Poly(azanediyladipoylazanediylhexane-1,6-diyl) is used to make nylon fibers, with the remainder going into technical or engineering resins (Palmer, 2005). Nylon fibers are commonly found in fabrics, carpets, and molded parts (Xiang et al., 2020). Poly(azanediyladipoylazanediylhexane-1,6-diyl), mainly the glass fiber reinforced grade, is widely used to make fire-safe polymers that pass flammability tests and wire fire tests in the electric industry (Kauffman, 2010; Rezvani Ghomi et al., 2020). Poly(azanediyladipoylazanediylhexane-1,6-diyl) is used in various applications due to its high mechanical strength, rigidity, temperature resistance, and chemical resistance (Nguyen et al., 2020a, 2020b; Owlia et al., 2019; Kim et al., 2019; Choudhari and Kakhandki, 2021).

### 2.2 Acrylonitrile-butadiene-styrene

It is commonly known as polycarbonate. It is a high molecular weight polymer with carbonate parts as connecting groups for diols with either aliphatic or aromatic systems. Aromatic polycarbonates are used widely, thus considered as significant engineering plastic. Aromatic polycarbonates are prepared by reacting phosgene with polycarbonate. In 1941 Pittsburgh Plate Glass Company (PPG) introduced the polycarbonate resin. The commercially available polycarbonate resin was manufactured which is an aliphatic cross-linkable liquid, CR 39 (Wehrmann, 2001). Polycarbonates are synthetic polymers derived from bisphenol A (BPA). It has high heat capability, optical clarity, and extreme hardiness. Polycarbonate is beneficial for manufacturing plastics and synthetic polymers. The phosgene process has been used for the production of BPA-based polycarbonates. Recently, various industries have developed environment-friendly synthesis processes. Instead of phosgene and chlorinated solvents, diphenyl carbonate, carbon monoxide, or carbon dioxide are used as alternatives (Takeuchi, 2012).

### 2.3 Poly(ethyl benzene-1,4-dicarboxylate)

It is commonly known as terephthalic acid. Poly(ethyl benzene-1,4-dicarboxylate) is widely applied in packaging industries (Baskaran and Sathiavelu, 2022). The global acceptance of poly(ethyl benzene-1,4-dicarboxylate) is in our daily usage of household materials because of its good compatibility with food, consumer products, medicine as well as high recyclability and strength (Amobonye et al., 2023). It is the most important polyester and its production reached about 30.3 million metric tons in 2017. The distinguishing feature of poly(ethyl benzene-1,4-dicarboxylate) is a clear, amorphous thermoplastic when it is rapidly cooled. When poly(ethyl benzene-1,4-dicarboxylate) cooled slowly or cold-drawn it became a semicrystalline plastic. Poly(ethyl benzene-1,4-dicarboxylate) is produced by polycondensation of ethylene glycol and terephthalic acid. The processes are known as injection molding, blow molding, and extrusion. In carbonated beverage packaging industries, poly(ethyl benzene-1,4-dicarboxylate) has a great role due to its lower gas saturation capacity compared to low-density polyethene, poly(1-phenylethene-1,2-diyl), and polylactic acid (Sin and Tueen, 2023). Poly(ethyl benzene-1,4-dicarboxylate) and related polyesters are difficult to plasticize because of their crystalline nature. Nylon is plasticized by using water. The poly(ethyl benzene-1,4-dicarboxylate) is also plasticized in presence of less water. During the formation of high-strength film and textile fiber, there are little or no external plasticizers used in it (Wadey, 2003). The polymer matrix of ethylene terephthalate monomers is used for the formation of poly(ethyl benzene-1,4-dicarboxylate) with alternating C_10_H_8_O_4_ units. Poly(ethyl benzene-1,4-dicarboxylate) is dense and semi-rigid or rigid therefore commonly used for carbonated drinks due to its strong gas and moisture blocker capacity (Paladhi et al., 2022).

### 2.4 Poly(propene)

Poly(propene) belongs to the thermoplastic group and is used in various industries. It is manufactured through the polymerization of propylene gas. Propylene is a gas obtained from petroleum hydrocarbons and propane at high temperatures. It is hard and rigid as well as having low density. It is resistant to environmental stress such as high temperature. Poly(propene) is isotactic, atactic, and syndiotactic as per the stereotactic structures out of which 95% isotactic structure is seen in commercial poly(propene) (Koerner et al., 2007). Poly(propene) is used in multilayered films because of its exceptional strength. It has little surface energy, low gas permeability, liquid permeability, and relatively simple processing. The opacity of poly(propene) is detrimental when used for packaging purposes. Its orientation breaks the spherical structure to get clear film. Moreover, orientation improves tensile properties when it is pulled. It also increases strength and the permeability of gases is also reduced (Calhoun, 2010). Poly(propene) added with flame retardants are used in electronics, construction, and transport. Antiblock and anti-slip agents are used to prevent the block formation in the films (Hansen et al., 2013). In automotive applications hindered phenolic or hindered phosphite process stabilizers and hindered amine light stabilizers are combined with poly(propene) to slow down the process of oxidation (Massey, 2007). The heat-stabilized poly(propene) is more compatible with steam sterilization, whereas un-stabilized poly(propene) may be degraded by heat. The degradation of poly(propene) may occur after three successive autoclaving (Lerouge and Simmons, 2012).

### 2.5 Poly(1-phenylethene-1,2-diyl)

Poly(1-phenylethene-1,2-diyl) is a liquid hydrocarbon commercially manufactured by Baden Aniline and Soda Factory (BASF) in 1930 from petroleum. Poly(1-phenylethene-1,2-diyl) is an aromatic polymer of styrene monomer. At room temperature, poly(1-phenylethene-1,2-diyl) is typically in solid form but it can be melted at elevated temperatures for molding or extrusion and then resolidified (Koerner et al., 2007). As per Myer (2011) produced amount of poly(1-phenylethene-1,2-diyl) was 1990 thousand metric tons, and 30 thousand metric tons were recycled. Poly(1-phenylethene-1,2-diyl) is mainly used in various forms such as solid, foam, and expanded poly(1-phenylethene-1,2-diyl) (Merrington, 2011). A modified form of poly(1-phenylethene-1,2-diyl) is high-impact poly(1-phenylethene-1,2-diyl) is bright white colored and biodegradable. For manufacturing limonene, it is used as a solvent. It is the perfect material for surgical and dental instruments and has no contrary effects when biological structure comes in close contact. High-impact poly(1-phenylethene-1,2-diyl) has excellent resistance and toughness at 210°–250°C temperature range (Thomas, 2021).

### 2.6 Poly(1,1,2,2-tetrafluoroethylene)

Poly(1,1,2,2-tetrafluoroethylene) is a fluoropolymer material known by its trade name, Teflon. Poly(1,1,2,2-tetrafluoroethylene) was described by Plunkett in 1938. It is a highly fluorinated saturated organic compound with unusual physical and chemical properties (Hanford and Joyce, 1946). The unique properties are nonreactivity, hydrophobicity, good insulation, and low coefficient of friction. It is most commonly used for cookware coating due to its nonstick properties (Radulovic and Wojcinski, 2014). Fluorine changes the property of poly(1,1,2,2-tetrafluoroethylene) material. The special properties of poly(1,1,2,2-tetrafluoroethylene) involve resistance to organic solvents, low friction surface, and nonstick nature. It is also resistant to electricity, adverse meteorological conditions, corrosion, and UV light. Poly(1,1,2,2-tetrafluoroethylene) is inactive, non-toxic, and compatible with human tissue therefore used in surgical instruments (Adtech, 2024).

### 2.7 Ethylurea

Ethylurea is a special polymeric material that is used in items like liquid coatings, elastic fibers, elastomers, insulators, integral skins, and making of foams. Ethylurea is a versatile group of synthetic heteropolymers that can be produced as thermoplastics, thermosets, elastomers, coatings, adhesives, and sealants (Gaytan et al., 2020; Khruengsai et al., 2022). It occurs in numerous forms with simple improvements made by Professor Dr. Otto Bayer and co-workers (Akindoyo et al., 2016). Researchers invented the di-isocyanate polyaddition technique for the formation of the ethylurea industry in the year of 1937. It is formed by the reaction between di-isocyanate and polyester diol (Delebecq et al., 2012). Ethylureas are of various types like rigid ethylurea foams, flexible ethylurea foams, ethylurea ionomers, etc. Rigid ethylurea foam is an energy-saving insulation material. Flexible ethylurea foams contain block copolymers and flexibility depends upon the phase separation between soft and hard segments (Cinelli et al., 2013). Thermoplastic ethylurea show enormous combinations of physical properties and processing applications. It is flexible and elastic and has good resistance power from abrasion and weather conditions. In ethylurea ionomers, ionic groups are present in the ethylurea backbone chain so the benefit is better dispersion in polar solvents, it also enhances hydrophobicity as well as thermal and mechanical properties (Jaudouin et al., 2012).

### 2.8 Polyethene

Polyethene is a repeating unit of ethylene (CH_2_) monomers having main characteristics like low cost, electric insulation, great chemical resistance, hardiness, elasticity, and thinness. The high-pressure processes used for the production of commercial polyethene are based on molar mass. Polyethene is classified as linear low-density polyethene, low-density polyethene, ultra-high-density polyethene, and high-density polyethene due to the discovery of metal compounds. The compounds based on Cr (Phillips catalyst) or Ti (Ziegler–Natta) have the ability of ethylene polymerization at less extreme conditions (Ronca, 2017). High-density polyethene is lightweight with good tensile strength and low-density polyethene is a chemical-resistant plastic material (Kumar et al., 2022). Polyethene can be transformed from thermoplastic to thermoset plastic (Plastic Europe, 2017). It is the polymer of ethylene with high molecular weight and a long chain of hydrocarbon (Shimao, 2001). Around 500 billion to 1 trillion polyethene carry bags are utilized worldwide for packaging and carrying various products. Worldwide highest number of plastic consumers is in Asia at 35% after that North America at 26%, Western Europe at 23%, Japan at 6%, and finally India at 5% (Ali et al., 2014).

In addition to these polymers, additives such as catalyst deactivators, plasticizers, colorants, reinforcements, nucleators, antistatic agents, flame retardants, anti-block, slip agents, and stabilizers are involved. These additives work for enhancing the functional properties of plastic products. The applications of these additives for increasing the properties of plastic polymers, such as catalyst deactivators play a role in neutralizing the remaining catalyst residues. Nucleators are used to reduce processing time and increase the clarity of resin. Colorants are used for developing various colored varieties of plastic material. Antistatic agents are used to permit the discharge of static electricity from the film or part.

## 3 Plastic pollution

Environmental pollution is a common thing on the earth. Plastic pollution is one of them, due to plastic accumulation in a particular area raising big health issues related to the terrestrial and marine environment. Unlimited use of plastic daily and thrown in open areas that become dumping sites for plastics and a huge number of plastics accumulate on the shoreline and in the deep ocean marine environment (Browne et al., 2008; Thompson et al., 2009; Barnes et al., 2009). The global annual production exceeded 359 million metric tons as per Pilapitiya and Ratnayake (2024), in 2023, plastic production reached 159 million metric tons worldwide (Liu et al., 2023). In the year 2020, plastic production was about 367 million metric tons and for 2019 it was 368 million metric tons during the COVID-19 pandemic (Kaul, 2021). Plastic production has surged from 1.7 million metric tons in 1950 to over 400 million metric tons by 2019 (Geyer, 2020). The worldwide plastic production is calculated as 400.3 million metric tons in 2022 (Rafey and Siddiqui, 2023). According to Plastic Europe (2021) global annual production of plastic has increased from 2 million metric tons to 368 million metric tons by 2019. It was around 335 million metric tons in the year 2015 (Plastic Europe, 2017). About 300 million metric tons of plastic was found to be produced every year (Plastic Europe, 2015). The report of Lederberg (2000) explained about 57 million metric tons of plastic is produced in the year, 2000 and approximately 53 kilo metric tons of plastic waste is expected to be released into the environment by 2030 (Borrelle et al., 2020) while the production of thermoplastic is projected to reach 445.25 million metric tons by 2050 (Soong et al., 2022). In European households and all areas of the packaging industry produced around 82.5 million metric tons of plastic waste per year in 2014 (Eurostat, 2017). The total 40% of plastic is used in Europe for food packaging (Marsh and Bugusu, 2007; ING Economics Department, 2019; Geyer et al., 2017). In the US 69.6 million metric tons of packaging waste is collected as solid waste (EPA, US, 2016).

Arya Mitra, an Earth5R volunteer from Dehradun took the initiative of plastic waste management and reported plastic products from the waste. Out of 246 collected plastic waste items, 150 were multi-layer packaging (MLP) products, making up 60.9% of the total. This was followed by 48 low-density polyethene (LDPE) items (19.5%), 19 tetra packs (7.7%). Nine high-density polyethene (HDPE) products (3.6%), and 5 poly(ethyl benzene-1,4-dicarboxylate) products (2.03%) (Earth5R, 2014).

Among the different methods for plastic waste management, different countries follow traditional strategies like incineration and landfilling. As per the UK supply chain composite waste report in 2015, nearly 98% of compound waste is buried inside landfill areas (Naqvi et al., 2018; Krauklis et al., 2021). In India till 2022, 9% of plastic was recycled from the 7 billion metric tons of plastic produced. Municipal solid waste is dumped in open areas. Around 1.5 million metric tons of plastic waste is produced yearly in India (Tiwari et al., 2023). It shows adverse side effects on human health and the environment (Sharholy et al., 2008). Plastic pollution is a highly challenging problem for the globe. Everywhere we see plastic in various forms such as polyethene or poly(propene) carry bags, and various kinds of wrappers used for packaging. These packaging materials need to be disposed of properly. During incineration of plastic material, a large amount of CO_2_ is released into the environment and the landfilling process pollutes groundwater.

Landfilling and incineration do not recover the material after the process so these are not recycling strategies today (Jang et al., 2020; Dong et al., 2015; Zhou et al., 2021). Some European countries have decided to prohibit landfills of plastic waste and expecting 10% reduction in municipal waste by 2030 (Shuaib and Mativenga, 2016). Only 10 and 14% of plastic waste is incinerated globally and 76% of plastic material is dumped in the environment or landfilling sites (Geyer, 2020). Around 10% of plastic is burned by the process of incineration (Jouhara et al., 2018). Due to incineration, large amounts of greenhouse gases are emitted into the environment such as nitrogen oxides, hydrogen sulfide, carbon dioxide, carbon monoxide, nitrogen, oxides, sulfur oxides, and other toxic substances like dioxins, furans, halogens, polychlorinated biphenyls, and fly ash. It harms to environment and creates various health issues (Chang, 2018; Verma et al., 2016). India produces around 70 metric tons of municipal solid waste per year and by 2030 it will reach approximately 165 metric tons. In the future, it could be reached around 436 metric tons by 2050 (Planning Commission Report, 2014). As per the report, 25 million tons of plastics are accumulated every year in the terrestrial and marine environment (Arutchelvi et al., 2008; Smith, 1964; Iiyoshi et al., 1998). The 6–7% of solid waste generated from municipal areas is used in India for compost formation, and the remaining amount of municipal solid waste is disposed of through landfilling (Annepu, 2012). Industries release hazardous materials and mixed with municipal solid waste creating a risk to human health (Alam and Perrone, 2013).

Single-used polyethene carry bags and other various types of plastic materials are thrown by people in an open environment in a particular area known as dumping sites. Municipality workers collect millions of metric tons of plastic material from the dumping sites and shift it to the garbage depot of the city daily. Water channels drown large amounts of plastic waste present in that area. A huge amount of plastic and pollutants from the cosmetic industry and household waste material drown by the drainage and river systems and finally enter the water reservoirs (Cole et al., 2011). As per the scientific report, coastal traveling, fishing, and marine industrialization are core sources of plastic waste generation in the ocean system (Veiga et al., 2016; Cole et al., 2011). Finally, the concentration of plastic in seashores, rivers, and estuaries gets increased (Maes et al., 2017).

### 3.1 Effect of plastics on plants and animals

During incineration the released organic gases and ashes damage plant structures and disturb wildlife when inhaled by different fauna. Sulfur dioxide, carbon dioxide, and other gases are released into the atmosphere during the melting of plastic and are responsible for global warming and acid rain (Shen and Worrell, 2024). Landfilling of plastic waste leads to the leaching of chemicals into the soil and affects life. In aquatic ecosystems, plastic ingestion is the main cause of intestinal blockage, and entanglement (Amobonye et al., 2021). The first encounter of plastics in wildlife in the form of plastic debris in the guts of seabirds, was reported in 1960 (Suaria et al., 2020). Plastics are fragmented further in the dumping sites and converted into pieces of various sizes. As per the Guidance on Monitoring of Marine Litter in European Seas, plastics are distinguished into macro-plastics (>2.5 cm), meso-plastics (0.5–2.5 cm), large micro-plastics (0.1–0.5 cm), small micro-plastics (<1 mm) and nano-plastics (<100 nm) (European Commission, 2013). Microplastics and nanoplastics are deleterious for natural ecosystems because they act as vectors for further contaminants (Besseling et al., 2014; Teuten et al., 2007) which is risky for the roots of plants for nutrient uptake (Engler, 2012). The micro and nano-size particles are the origin of soil pollution (Horton et al., 2017). Irrigation, plastic mulching, soil alterations, and flooding, are factors that pollute soil by adding microplastic (Dris et al., 2015; Eerkes-Medrano et al., 2015; Steinmetz et al., 2016; Majewsky et al., 2016; Mahon et al., 2017; Kim and An, 2019). Plastics are degraded due to sunlight into toxic particles which contaminate soil and water sources. These toxic particles are unknowingly ingested by aquatic animals (Denuncio et al., 2011). In the aquatic environment, polyethene waste is the main cause of intestinal obstruction in fishes, birds, and aquatic animals (Denuncio et al., 2011; Spear et al., 1995). As per the report, 267 species including all mammals, 86% of sea turtles, and 44% of seabirds were affected due to plastic pollution (Coe and Rogers, 1997). Plastic carry bags sometimes lead to the death of terrestrial animals like cows and goats by blocking the digestive tract (Singh, 2005). The polyethene is not digested in the stomach of the animals and after their death same plastic material is eaten by the next tropic-level animals. The undigested polyethene hampered parts of the digestive system. The swallowed polyethene blocks the opening between the omasum and reticulum and causes animal death (Singh, 2005).

To control the plastics pollution, the government agencies like pollution control boards, and other organizations including NGOs have taken initiatives. In Nairobi on 2nd March 2022, the UN Environment Assembly (UNEA-5) and heads of state, minister of environment, and other UN member representatives assembled and sanctioned the “End Plastic Pollution” and forged an international legally binding agreement in, 2024 (UNEP, 2022). In India, the Central Pollution Control Board banned the production, dumping in open areas, and selling of carry bags below 20-micron thickness (Anonymous, 2006). The Maharashtra state of India also prohibited production and selling of plastic carry bags less than 50-micron thickness. However, plastic carry bags of less than 50-micron thickness are being used illegally in the market, leading to health issues for humans and living organisms.

There are plastic disposal strategies under practice such as landfilling (65%), incineration (25%), and recycling (10%) which have their own limitations. The limitation of landfills is that they may exacerbate land shortages (Kong et al., 2017). It also contaminates water bodies during the monsoon season (Alabi et al., 2019). The landfilling of plastic waste is responsible for soil contamination or other words soil pollution (Rajmohan et al., 2019). When we use the recycling method it produces pollutants by breaking the plastic material. This process is not cost-effective and hampers the quality of the products hence the value of recycled products is less (Meenakshi, 2018). Researchers said that incineration is not a preferred method of disposal of plastic waste management because it can result in the emission of dioxins and other atmospheric hazardous pollutants that contribute to global warming (Niaounakis, 2013).

Plastic carry bags also block the drainage lines and a flood-like situation is developed in rainy season due to which multiplication of the vectors like mosquitoes occurs leading to the emergence of pathogens. After fragmentation of the plastic materials microplastic is generated in the soil which affects the population of plants and animals (soil dwellers). The plants and animals pass these microplastics to higher animals and human being which creates serious health issues. All type of plastics finally accumulates in the marine environment through channels, streams and rivers and affects marine animal health. Used plastic carry bags also get floated in marine water at the coastal area leads to health hazards of marine animals. Ultimately affected marine animals are hampered due to the malfunctioning of their digestive tract (Muthukumar and Veerappapillai, 2015).

## 4 Methods of plastic waste management

In some countries, the production of biodegradable plastics, fuel production from the plastic, and mixing plastic with bitumen for road construction are the ways to manage plastic pollution. The methods are used for plastic disposal have their advantages and disadvantages. These are discussed as follows.

### 4.1 Landfilling

Landfilling is a common method of plastic waste management, approximately 10% of plastics generated from households are dumped in landfills (Hopewell et al., 2009). In Western Europe 65% (8.4 million tons per annum) of plastics recovered from overall household wastes were sent to landfill in 1999 (Association of Plastics Manufacturers in Europe, 2002). In Germany 20% France 37% and in England 60% of municipal waste is disposed of in landfills (European Environment Agency, 2007). Landfilling raises environmental and public health problems because of toxic chemicals leached at landfill sites (Miller, 2005). Therefore, the government policy, Landfill Directive European Commission 1999/31/EC in the UK reduced the amount of waste disposed to landfills. Thus, public health and environmental pollution can be reduced by banning plastic landfills. However, disintegrated plastic byproducts and additives persist in the environment for a long time which can contaminate soil and groundwater (Oehlmann et al., 2009; Teuten et al., 2009). Landfilling with plastics includes lighting up methane partially responsible for climate change as well as contaminating water, soil and affecting wildlife (Kedzierski et al., 2020).

### 4.2 Degradation

There are number of methods which can be followed for the degradation of plastic with the help of radiations, strong oxidizing and reducing agents. Generally, photodegradation, thermo-oxidative degradation, and biodegradation methods are used for plastic waste management (Shah et al., 2008). Tian et al. (2021) highlighted degradation methods that can be categorized on the basis of their mechanisms such as photothermal degradation, ozone-induced degradation, catalytic degradation, and biodegradation. Degrading plastic material through various means becomes one of the alternatives to deal with plastic pollution (Karaduman, 2002; Griffin, 1994).

#### 4.2.1 Photodegradation

Light is the main source for photo-oxidative degradation of plastic material. UV (290–400 nm) and visible light initiate the degradation of polymers (Ranby, 1989; Jensen and Kops, 1980; Sheldrick and Vogl, 2004). UV radiations cleave the C-C bond because of its available energy (Mark et al., 1986). Degradation of polymers occurs mainly in the ether parts of the soft segments, where photo-irradiation produces ester, aldehyde, formate, and propyl end groups (Nagai et al., 2005). The most damaging UV wavelengths are around 300 nm and 370 nm for polyethene and poly(propene). Photodegradation changes the physical and optical properties of the plastic by changing its yellowish color (Torikai, 2000). Direct UV-initiated photolysis of C–C and C–H bonds takes place on exposure of UV. UV radiations of 320 nm and 290 nm are equivalent to the dissociation of the C–C bond (375 kJ/mol) and C–H bond (420 kJ/mol) so direct photolysis of the C–C and C–H bond is possible by UV radiation (Plotnikov, 1988). Low-density polyethene has been reported to undergo photo-oxidation at a higher rate compared to high-density polyethene because it contains a greater number of reactive branch points (Craig et al., 2005). In polyethene, photo-degradation sharpens peaks for ketones, esters, and acids in the infrared spectrum. Poly(propene) also shows this effect but is more resistant. For poly(ethyl benzene-1,4-dicarboxylate), photo-oxidation forms hydroperoxides by oxidizing CH_2_ groups near ester linkages, leading to various photoproducts through multiple pathways (Fotopoulou and Karapanagioti, 2017). Synthetic polymers absorb solar ultraviolet radiation that leads to photolytic, photo-oxidative, and thermo-oxidative reactions that result in degradation of plastic (Gugumus, 1993; Andrady, 1997). The degradation of plastic products induced by solar UV radiation has been increased by adding suitable additives to these polymers (Cataldo, 2001; Andrady et al., 2003). Photodegradation rates decreased in the order of poly(1,1,2,2-tetrafluoroethylene), followed by polyesters, and then polyamides (Min et al., 2020).

#### 4.2.2 Thermal degradation

Thermal degradation of polymers occurs by random and chain degradation mechanisms (depolymerization reaction) initiated by heat and UV light (Teare et al., 2000). In the thermal decomposition of the depolymerization reaction, it is not necessary to start from the end of the macromolecule, but the imperfection of the chain structure (initiator fragment or peroxide or ether bond) forms a weak link from which the depolymerization begins. A large number of synthetic polymers depolymerize at high temperatures (Goodings, 1961; Kamerbeek et al., 1961). Thermal degradation of plastic above 200°C leads to chain scission and largely depends on impurities like unsaturation sites, head-to-head units, etc. (Ramis et al., 2004).

The limitations of these methods involve expensive setup of instruments than the other methods. It produces harmful gases and pollutants in the atmosphere and causes health-related problems. After incineration of plastic material, ash is emitted into the environment and harms living organisms (Netzer et al., 2021).

#### 4.2.3 Chemical degradation

It involves molecular-level changes, such as bond cleavage or the oxidation of long polymer chains, resulting in the formation of new molecules with shorter chain lengths. It is essential to consider the potential environmental hazards posed by the soluble chemical byproducts of plastic degradation (Gewert et al., 2015). Chemical degradation at near-ambient environmental temperatures generally involves hydrolysis or oxidation (Andrady, 2011). The backbone chains of polyethene are made solely of C–C single bonds, which do not easily undergo hydrolysis and are resistant to photo-oxidative degradation because they lack UV–visible chromophores. However, impurities or structural defects that develop in polyethene during manufacturing or subsequent weathering can act as chromophores (Grassie and Scott, 1988; Hartley and Guillet, 1968). Polyethene may have a small number of unsaturated (C═C) bonds in the main chain or at the end of the chain. These sites are easily oxidized by ozone (O_3_), nitrogen oxides (NO*x*), or other tropospheric radicals, often forming highly unstable hydroperoxides that subsequently convert into more stable, UV-absorbing carbonyl groups (Rabek, 1995).

#### 4.2.4 Biodegradation

**Figure 1:**
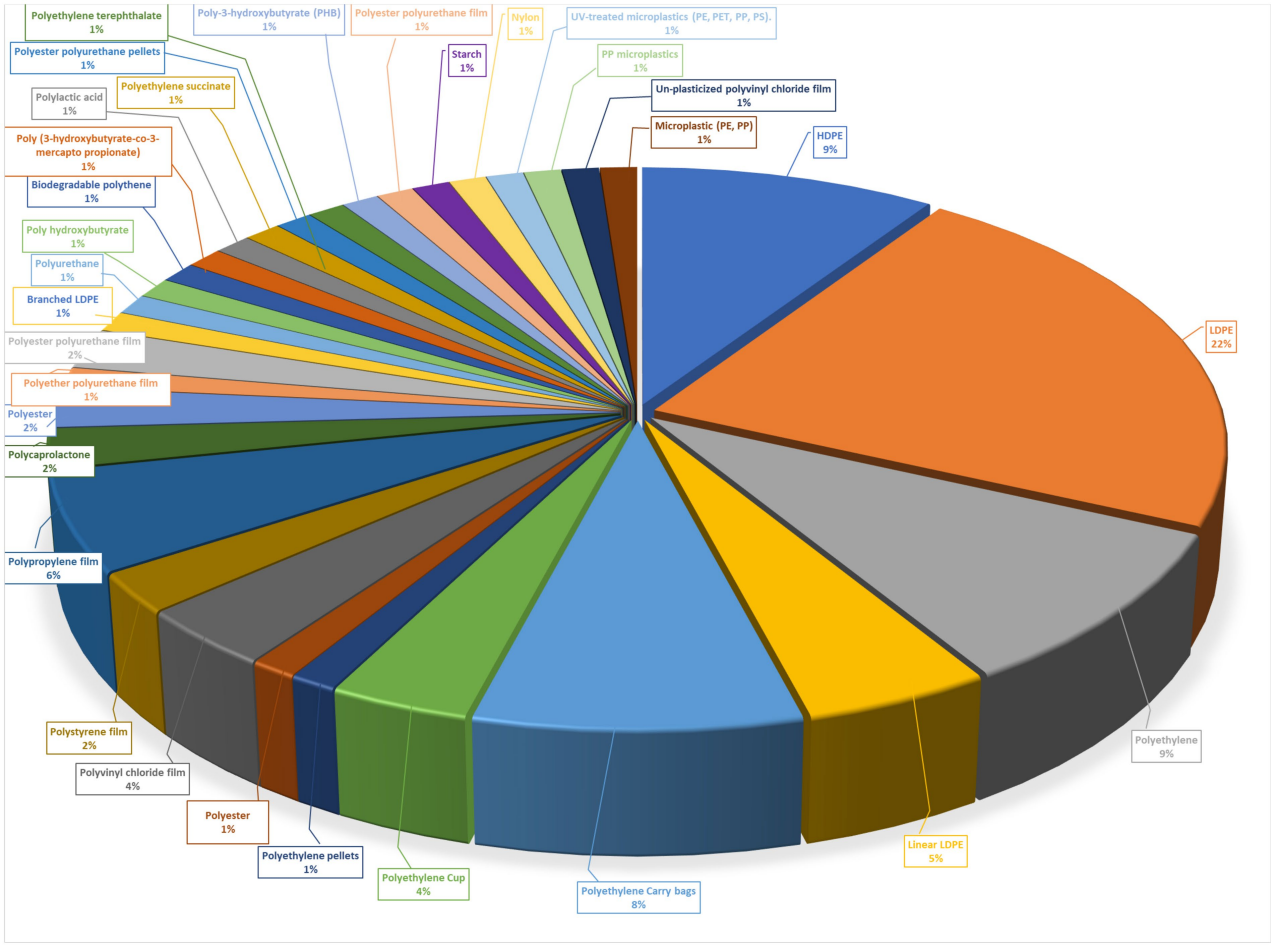
Types of plastics used for biodegradation.

Although biodegradation is a slow process it is cost-effective, economical, and eco-friendly (Sangale et al., 2012). Microbial degradation involves the use of bacteria, fungi, and actinomycetes. Polyethene has a slow or least degradation rate in the natural environment and requires 1,000 years for its degradation because microbes are unable to degrade plastics due to a lack of plastic-degrading enzymes present for a short period (Corcoran et al., 2009; Mueller, 2006). A comparative analysis of paraffin and polyethene was recorded first time when bacteria utilized polyethene as the carbon source for growth (Jen-hou and Schwartz, 1961). Bacteria can degrade low-density polyethene up to 48,000 molecular weight (MW) and later degrade high-density polyethene up to 93,000 molecular weights, due to the presence of short-chain oligomers and its molecule consisting of relatively few repeating units (Albertsson and Banhidi, 1980). The high accumulation of polyethene carry bags and other plastic materials at dumping sites leads to several health problems that biodegradation can overcome (see Figure 1).

**Figure 2:**
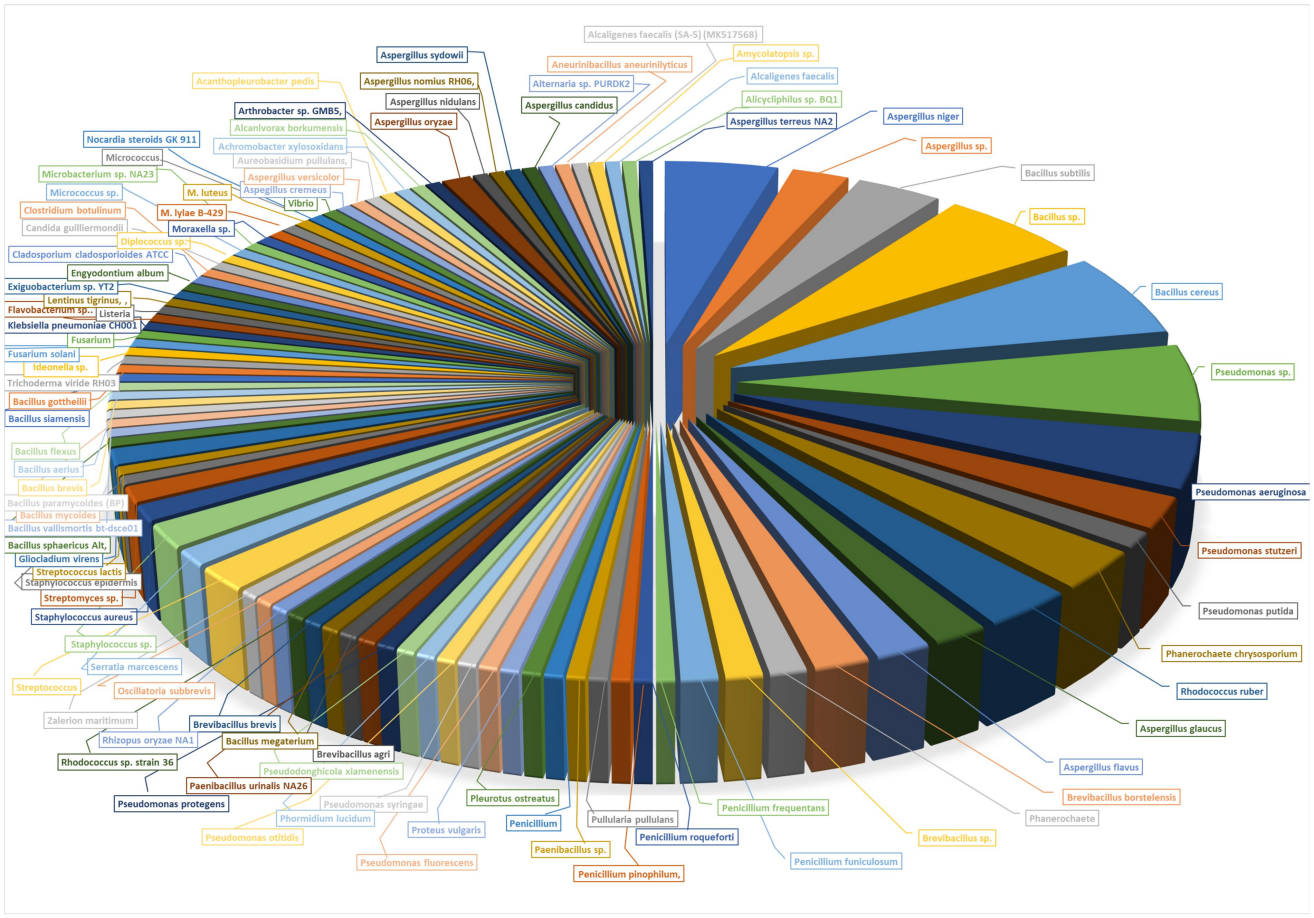
Fungi used for biodegradation of plastics.

In biodegradation, microorganisms adhere to the plastic surface, grow and form biofilms by using the polymer as a carbon source, enzymatically cleave the polymer into shorter-chain molecules, and ultimately degrade it into low molecular weight oligomers, dimers, and byproducts like CO_2_, water, or methane (Bhardwaj et al., 2013; Arutchelvi et al., 2008). Biodegradation of plastics could be achieved by microorganisms like fungi, bacteria, and algae in the ecosystem (see Figure 2). The biodegradation mechanism undergoes colonization, biodeterioration, biofragmentation, assimilation, and mineralization, eventually converting plastics into CO_2_ and H_2_O (Liu et al., 2023). Biodegradation, the breakdown and assimilation of materials by microorganisms or their byproducts, offers a promising method for the clean and gentle degradation of plastics (Leja and Lewandowicz, 2010). Studies have shown that organisms secrete degradative enzymes, which target the polymer backbone under environmentally neutral conditions with minimal energy input (Ronkvist et al., 2009). Naturally occurring microorganisms in diverse environments like soil, compost, or marine habitats have evolved to degrade plastic over time and with prolonged exposure, allowing them to metabolize it as an energy source (Emadian et al., 2017). Various bacterial and fungal species have been identified with enzymes that act on polymers like poly(propene), polyethene, and poly(ethyl benzene-1,4-dicarboxylate). For example, three bacterial strains from the Arabian Sea, i.e., *Kocuria palustris* M16, *Bacillus pumilus* M27, and *Bacillus subtilis* H1584, whereas soil-derived fungal strains *Aspergillus niger* and *Penicillium pinophilum* caused slight weight loss in polyethene films (Polk et al., 1999). Biodegradation has been observed with *Aspergillus flavus* on polyethene (Zhang et al., 2020), various *Cladosporium* species, and others on ethylurea (Alvarez-Barragan et al., 2016; Russell et al., 2011). Certain fungal cutinases and lipases are used for hydrolyzing poly(ethyl benzene-1,4-dicarboxylate) (Carniel et al., 2017). Additionally, *Coniochaeta hoffmannii* and *Pleurostoma richardsiae* have partially degraded poly(propene) (Porter et al., 2023). Fourier transform infra-red (FTIR) and Raman spectroscopy revealed that certain strains modify the properties of plastic polymers: *Cladosporium* sp. EXF-13502 affects polyamide, *Rhodotorula dairenensis* EXF-13500 impacts poly(propene), *Rhodotorula* sp. EXF-10630 influences low-density polyethene, and *Wickerhamomyces anomalus* EXF-6848 alters poly(ethyl benzene-1,4-dicarboxylate) (Cernosa et al., 2024). Microorganisms can provide enzymes that break down plastics into oligomers and monomers. These can then be reused to create new polymers, contributing to a truly circular economy for plastics (Danso et al., 2019; Tournier et al., 2020).

### 4.3 Recycling

Recycling of plastic generally comprises processes such as melting, shredding, or granulation of plastics. Feedstock recycling includes pyrolysis, catalytic conversion, depolymerization, and gasification. These processes are used for hydrocarbon formation from plastic waste. The recycled polymers are then converted into refined chemicals or fuels prepared from hydrocarbon products. Large amounts of plastic waste are recycled (Tukker, 2002; Chiellini et al., 2003; Contat-Rodrigo and Ribes-Greus, 1998). Recycling plastic helps to minimize pollution as there is no new manufacturing of plastic (Basta and Johnson, 1989). On the basis of the products generated recycling is divided into primary, secondary, tertiary, and quaternary types. Primary recycling involves the straightforward recovery of uncontaminated scrap plastic for reuse in similar applications. Although this method is low-cost, it is limited by a finite number of reuse cycles and is restricted to industrial materials with minimal contamination (Al-Salem et al., 2009). Secondary recycling of thermoplastics is a more complex process that involves cleaning, melting, and remolding the plastics into other forms. Only 6 to 26% of plastics are recycled (Alimi et al., 2018). These new forms, such as building or packing materials, have less stringent requirements for physical robustness. Even though virgin resins can be blended in plastic to improve physical properties, this inevitably results in compromised material homogeneity and restricts the range of downstream applications (Singh et al., 2017).

In tertiary recycling poly(propene), polyethene, poly(ethyl benzene-1,4-dicarboxylate), and poly(1-phenylethene-1,2-diyl) at 500°C are used for pyrolysis and converted into liquid fuels (Trivedi et al., 2016; Karamanlioglu et al., 2017; Emadian et al., 2017). The conversion of post-consumer plastic waste into oil by direct liquefaction and pyrolysis can be the methods for recycling which are followed by hydroprocessing of the pyrolysis liquids (Shah et al., 1999). In quaternary recycling, energy is obtained from plastic through cold plasma pyrolysis which can convert plastic into hydrogen, methane, and ethylene (Kumar, 2021). Cement-based composite is made from post-consumer waste plastics by specific mechano-chemical treatment (Naik et al., 1996). Poly(ethyl benzene-1,4-dicarboxylate) plastic is used as a new type of pellet fuel to recycle plastic waste and improve fuel efficiency (Sandro et al., 2019). The physical method of handling plastic is to convert it directly to granulate or to modify and reuse it as new plastic material. The recycled plastic is free from contamination, enabling it to be reused for comparable uses (Liu et al., 2023).

Approximately 6,300 metric tons of plastic waste was produced in 2015 where 9% was recycled, 12% burned, and 79% accumulated in dumping sites in an ecosystem (Wojnowska-Baryła et al., 2022). These methods require specific environmental conditions. During the recycling of plastic waste, hazardous chemicals such as polycyclic aromatic hydrocarbons (PAHs), dioxins, furans, persistent organic pollutants (POPs), volatile organic compounds (VOCs), and heavy metals are released from the combustion sites of plastic material. Besides the type of polymer, degradation rates are influenced by the properties of the plastic material, including the surface area-to-volume ratio and the presence of antioxidants and other stabilizers added during formulation and compounding to enhance durability (Arp et al., 2021).

Plastic waste degradation can be enhanced by a process where polymers react with nitrogen oxides and dioxygen under relatively mild conditions. Poly(1-phenylethene-1,2-diyl) produces a mixture of benzoic and nitrobenzoic acids. Both high-density and low-density polyethene yield mixtures of short-chain α and ω-diacids, such as adipic acid, in substantial quantities. Nylon-6,6 can form a mixture of acids (Pifer and Sen, 1998). Polymers from condensation polymerization, like poly(ethyl benzene-1,4-dicarboxylate), can be depolymerized back into their monomers. In contrast, polyolefins like poly(propene) and low-density polyethene, made via addition polymerization, cannot be similarly broken down and are better suited for pyrolysis (Al-Salem et al., 2017). The chemical recycling targets converting the polymer back into its original monomers or oligomers, with easily removable byproducts. Methanolysis and glycolysis are the most commercially advanced methods for this process. Methanolysis of poly(ethyl benzene-1,4-dicarboxylate) is conducted at high temperatures (180–280°C) and pressures (2–4 MPa) using transesterification catalysts like zinc acetate, resulting in dimethyl terephthalate (DMT) and ethylene glycol (EG) (Scheirs, 1998). Glycolysis of poly(ethyl benzene-1,4-dicarboxylate) is commonly used commercially, where prolonged treatment with ethylene glycol produces bis(2-hydroxyethyl) terephthalate (BHET), a substrate for conventional poly(ethyl benzene-1,4-dicarboxylate) synthesis, similar to DMT (Brandrup and Brown, 1996).

## 5 Economics of plastic waste management

The methods and their relevant expenditure are shown in Table 1.

**Table 1:** Cost-effectiveness of various processes for plastic waste management.

Sr. No.	Methods used	Expenditure	References
1	Collecting, transporting, treating, and disposing	Most urban local bodies spend $6–18 per metric ton of waste	Krishnan (2021)
2	Recovering recyclables and using landfills	Likely to cost $19 per metric ton	Krishnan (2021)
3	Incineration	Costs nearly double than landfills, $36–38 per metric ton	Krishnan (2021)
4	To collect, sort, dispose and recycle the plastic waste generated	It costs more than US $32 billion per year	Gikandi (2021)
5	Governments, NGOs, and concerned citizens undertaking clean-up activities to remove the waste	US $15 billion per year	Gikandi (2021)
6	Recycling 1 metric ton of plastic	It saves 13.8 barrels of oil, 5,744 kWh of energy, and 810 cubic feet of landfill space. It’s a great cost when 1 metric ton of plastic is not recycled	Lucro (2023)
7	Recycling 1 metric ton of high-density polyethene (HDPE) and 1 metric ton of low-density polyethene (LDPE) plastic types	It prevents 2.64 metric tons of CO_2_ equivalent emissions from entering the atmosphere, including 72,976 MJ of energy, and save 24 m^3^ of water in India	Lucro (2023)
8	Plastic waste management in China as scenarios of business as usual (BAU) and current policy scenario	For the business as usual net economic cost reached US $1.22 billion in 2020 and US $2.62 billion by 2035 and current policy scenario (CPS)-US$120 million by 2035	Sun et al. (2022)
9	Operating plastic waste management	In the U.S. the net cost burden is estimated at US $660 million annually	Anonymous (2023)
10	Canada’s plastic waste management system	Loss nearing $8 billion per year and is anticipated to escalate to over $11 billion by 2030	Anonymous (2023)

### 5.1 Challenges for plastic waste management

#### 5.1.1 Time for degradation

Plastic bag degradation times typically range from 10–20 years to 500–1000 years. For “plastic” bottles, the reported degradation time ranges from over 70 years to as long as 450 years. Some media sources have claimed that “plastics” do not degrade at all. For instance, a recent study revealed that poly(1-phenylethene-1,2-diyl) breaks down much more quickly under sunlight exposure than previous estimates of thousands of years suggested. Some studies indicate that plastic sheets can fragment due to solar radiation on beaches within months to a couple of years, whereas poly(ethyl benzene-1,4-dicarboxylate) bottles can remain intact for over 15 years on the sea floor (Fotopoulou and Karapanagioti, 2017). The use of catalysts, such as bentonite clays, can prioritize the production of primarily liquid pyrolysis oils, which are suitable for engine fuels (Sharuddin et al., 2016).

#### 5.1.2 Combining strategies for plastic waste management

Without sunlight, polyethene undergoes thermal oxidative degradation at negligible rates at temperatures below 100°C (Gardette et al., 2013). Conditions for biodegrading plastics are mild and products are non-polluting, but the process is very slow. From the results, a single method for treating plastics has certain limitations, and combining different methods has become a hot topic of study. Moreover, combining biodegradation and photodegradation of plastics is also an effective way to improve the degradation efficiency of plastics (Liu et al., 2023).

#### 5.1.3 The ideal schedule for plastic degradation

The natural plastic degradation process is biodegradation through microbes like bacteria, fungi, and algae (Rutkowska et al., 2000). Biodegradation is the most efficient and best alternative method for the plastic degradation. It is a natural process in which microbes are an important source for the degradation of plastics. Microbes are attached to the surface of the polyethene and utilize the carbon source and grow. However, the rate of degradation was less and required more time for biodegradation. The biodegradation rate depends upon the types of microbes. Some fungi and bacteria can degrade plastic at an efficient rate by providing a specific environment and maintaining pH.

In polyethene degradation, microbes are attached to the surface of the polyethene like *Streptomyces viridosporus* T7A, *Streptomyces badius* 252, and *Streptomyces setonii* 75Vi2 are the strains of bacteria that are used for the degradation of polyethene. Additionally, some wood-degrading fungi produced extracellular enzymes that play a role in polyethene degradation (Iiyoshi et al., 1998; Pometto et al., 1992; Champagne and Ramsay, 2005). Bacteria and fungi change the structure of chemicals through metabolic or enzymatic action in the process of biodegradation (Muthu, 2014). The investigated fungi which are able to degrade polyethene for 60 days of incubation at room temperature with continuous shaking conditions (Sangale et al., 2019a).

### 5.2 Assessment of polyethene degradation

Determination of polyethene degradation by various methods and analytical techniques. The topographical level of polyethene degradation like scission as well as attached microbes on the surface is analyzed by using scanning electron microscopy (SEM) (Spear et al., 1995). The microbial degradation of the polyethene material is analyzed by Fourier transform infrared spectroscopy (FT-IR), and the identified compounds map available on the sample surface and documented through a collection of a large number of Fourier transform infrared spectra which is helpful for the identification of compounds (Prati et al., 2010). To check the physical changes of the polyethene several parameters are used such as the tensile strength, percentage of weight loss, and elongation after microbial application. The polyethene degradation products are also characterized using various techniques such as high-performance liquid chromatography (HPLC) and gas chromatography-mass spectrometry (GC-MS) (Sangale et al., 2019b).

## 6 Conclusion

Plastic material is extremely useful in our daily routine for domestic to industrial needs such as wrapping goods, food material, medicine, scientific instruments, agriculture, etc. Plastics are lightweight and have good strength for carrying material so their usage is increasing gradually and its management is a big issue all over the world. Annually millions of tons of plastics are being utilized and thrown in open dumping sites. Deposition of huge municipal wastes containing plastics accumulated in the terrestrial and marine environment. Most of the animals feed on the plastic accumulated area along with fodder thus they consume plastics indirectly. Finally, a large number of terrestrial and marine animals are died due to intestinal blockage caused by plastic accumulation. There is a severe problem due to plastic pollution and we need to resolve this problem. The disposal and degradation are the most essential processes for substances in the matter. The cycle of disposal and degradation can be explored to tackle the problem of plastic pollution. Most scientists and researchers are working on this problem and trying to find out the most efficient plastic degradation method. Different polyethene degradation methods are there with their merits and demerits, however, an economical and eco-friendly method of biodegradation is needed. By combining the possible strategies, it can be practiced to resolve the degradation of plastic. The microbes utilize the carbon source from plastic for their growth by releasing extracellular enzymes to degrade plastic. However, the complete reaction mechanisms of such enzymes need to be investigated. The exploration of the microbes, associated with plastic degradation, is essential in order to find out the efficient strains which can be cultivated to a large extent with minimum expenditure. These are to be scaled up and commercialized for integrated methods of degradation of plastic waste.
